# Multiculturalism and Compassion: Responding to Mental Health Needs Among Refugees and Asylum Seekers: Comment on "A Crisis of Humanitarianism: Refugees at the Gates of Europe"

**DOI:** 10.15171/ijhpm.2019.77

**Published:** 2019-09-18

**Authors:** Emily Satinsky, Theodoros A. Filippou, Antonis A. Kousoulis

**Affiliations:** ^1^Center for Global Health, Massachusetts General Hospital, Boston, MA, USA.; ^2^Medical School, University of Athens, Athens, Greece.; ^3^Mental Health Foundation, London, UK.

**Keywords:** Global Mental Health, Health Services,, Cultural Diversity, Refugee Crisis, Europe

## Abstract

As Fotaki (2019) argues, the current political climate in Europe is threatening principles of humanitarianism, particularly among refugees and asylum seekers. This commentary builds on that argument, with a spotlight on mental health and culturally relevant service design. By addressing some of the barriers faced by refugees and asylum seekers in accessing mental healthcare, we can address inequalities and develop compassionate societies.

In “A crisis of humanitarianism: Refugees at the gates of Europe,” Fotaki argues that, despite our shared vulnerability and capacity for suffering, the political climate in Europe is undermining our universal right to protection.^1^ This case is particularly relevant to forced migration, with hostility against economic migrants, refugees, and asylum seekers; concerns around security and multiculturalism; and right-wing populist rhetoric increasing across Europe.^2^

This is a deeply moral argument with important public health implications. The sudden, forced change in individuals’ position in a global society creates pockets of inequality and jeopardises health. It is vital that we return to the concept of humanitarianism and recognise that as a human race, we are obligated to provide compassion and social solidarity to all individuals, with a specific focus on those most in need. This has been highlighted in the United Nations Special Rapporteur Report on the right of everyone to the enjoyment of the highest attainable standard of physical and mental health.^3^ Implementing a human rights-based approach to mental healthcare is particularly valuable,^4^ especially because mental health and well-being is shaped and supported by the wide-ranging characteristics of our social, economic, and physical environments.

## Understanding the Problem

Amidst the stress and upheaval endured in their host country, during migration, and throughout resettlement, refugees and asylum seekers face sudden changes in living conditions and dramatic cultural, social, and economic disruptions. These factors can compound with one another, increasing the likelihood of refugees and asylum seekers experiencing distress.^5^ Furthermore, the migration process can lead to elevated rates of post-traumatic stress disorder and common mental disorders including depression and anxiety.^6^ While rates vary by study, current estimates put the prevalence of post-traumatic stress disorder among refugees at around 15%^7^; this compares to a 1.1% prevalence estimated among non-refugee populations.^8^ Mental health problems can be magnified by unemployment, the absence of familial support, and difficulties navigating the asylum process.^9^

## Identifying Solutions: Accessible Care

To address high rates of distress and mental health problems among refugees and asylum seekers, accessible care is needed. This may include programmes that address general well-being (ie, preventive programmes that promote self-management; support groups for the re-establishment of social networks^5^), as well as specialist care (ie, psychotherapy, including manualized psychotherapeutic packages^10^; psychotropic drugs) for more severe cases. However, refugees and asylum seekers face barriers in accessing these services.^11,12^ While the literature reports distress attributable to displacement and loss, and a subsequent higher prevalence of mental health disorders among this population,^13^ there remains a discrepancy between need and help received. Under-utilisation of mental health services could indicate structural, cultural, or linguistic barriers faced more broadly by these populations. While all migrants face access barriers, refugees and asylum seekers are particularly susceptible to these challenges as a result of the unique trauma associated with their migration experiences.

A systematic review on access to mental healthcare among refugees and asylum seekers in Europe further reveals the inadequacy of service provision to this population.^14^ At the individual-level, common access barriers include differences in help-seeking behaviours, limited psychoeducation, lack of service awareness, language barriers, and cultural stigma towards mental illness.

Compounding individual-level barriers to care, the influx in refugee and asylum-seeking populations across Europe puts stress on national health services. Thus, at the service -level, access is further challenged by long wait lines, lack of specialist providers, and limited availability of interpretation services. Additionally, as many refugees and asylum seekers present at emergency departments and primary care settings with psychosomatic symptoms, they may be misdiagnosed by providers without backgrounds in cultural idioms of distress.

Although there is some emergency legislation requiring service provision for most at-risk groups, as described by Fotaki,^1^ many individuals go without care. In light of existing barriers to care, it is an urgent priority to address and improve mental healthcare for refugees and asylum seekers. According to the Migrant Immigration Policy Index from 2015, only six EU countries were rated ‘favourable’ with regard to health policies for migrants.^15^ Further policy analysis should be established to better understand how national governments and health services support responsiveness to psychiatric needs. By developing stronger policies and implementing innovative services that address access barriers, we can ensure equitable access to mental healthcare.

## Reforming Early Intervention

To improve access to quality and humane mental healthcare, healthcare systems should implement trauma-informed frontline services,^16^ as well as training programmes for frontline primary care providers.^17^ As cultures differ in expressions of mental health,^18^ and considering the high rates of somatisation and misdiagnosis among some refugees and asylum seekers,^19^ training should focus on the cultural, ethnic, linguistic, and experiential manifestations of distress common to different populations.^20^ To encourage sensitive and compassionate care, training programmes should teach providers about variant expressions of mental distress, while simultaneously encouraging providers to reflect on their own backgrounds and culturally-shaped assumptions. Furthermore, integrated care and improved communication channels across health and social services, and within and across countries, are important next steps.^21^

In addition to training programmes among frontline primary care providers, there is a need for improved psychoeducation among social workers, teachers, and community practitioners. Other structural barriers can be addressed through outreach, collaboration with interpreters, self-referral options, and expanded opening hours. Telepsychiatry and online services which allow individuals to talk with professionals who speak their language can be implemented to navigate language barriers. A systematic review from 2017 demonstrated efficacy of electronic approaches to psychotherapy; mobile phone-based technologies acceptably and feasibly targeted a number of mental health outcomes.^22^

Furthermore, in developing mental health services that are acceptable and appropriate for diverse cultural groups, we can look to the evidence from global mental health. A move towards ‘task shifting,’ particularly in community-based settings in destination countries, could build capacity while concurrently addressing access barriers around stigma and acceptability. Employing lay providers and ethnic minority staff including individuals from like cultural backgrounds, and providing training on evidence-based interventions and culturally appropriate care that takes individuals’ unique experiences into account can increase acceptability.^23^ For example, the STRENGTHS program for Syrian refugees in Europe and the Middle East has recommended task shifting mental health service provision for refugees and asylum seekers. The programme proposes employing lay providers and digital programmes to implement Problem Management Plus, an evidence-based intervention for common mental disorders.^24^ Similar strategies, with cross-cutting support from other European countries, should be developed and implemented.

## Concluding Thoughts

Mental health problems are still clustered around groups who face the greatest socio-economic inequalities, including refugees and asylum seekers; thus, it is important that we continue to drive this conversation forward. We must rigorously evaluate existing programmes and develop and implement innovative, trauma-informed, and culturally relevant services that effectively address the mental health needs of this population.

But perhaps above all, we need to return to the principles of humanitarianism, and use the ongoing crisis as an opportunity to shape compassionate societies and drive innovation in service design. To realise everyone’s right to good mental health, European nations need to ensure that refugees and asylum seekers themselves are given the opportunity to realise their potential.^3^ Despite enormous challenges faced by this population, refugees and asylum seekers can overcome these obstacles, rediscover an equilibrium, and become productive members of the communities in which they resettle. With increased care to building support systems and treating these groups with compassion, we can enable resilience among refugees and asylum seekers, allowing individuals to contribute in meaningful ways. It is of the utmost importance that the most at-risk across society are given the support and protection they require, and good well-being is not treated as a privilege.

## Ethical issues

Not applicable.

## Competing interests

Authors declare that they have no competing interests.

## Authors’ contributions

AAK conceptualised and ES and AAK framed the paper. ES led on data collection and preparing the initial draft. All authors further drafted and revised the work, assessed it for important intellectual content, and gave final approval prior to publication.

## Authors’ affiliations

^1^Center for Global Health, Massachusetts General Hospital, Boston, MA, USA. ^2^Medical School, University of Athens, Athens, Greece. ^3^Mental Health Foundation, London, UK.
